# A tumor acidity activatable and Ca^2+^-assisted immuno-nanoagent enhances breast cancer therapy and suppresses cancer recurrence†

**DOI:** 10.1039/d0sc00293c

**Published:** 2020-06-29

**Authors:** Yanhua Li, Shaohua Gong, Wei Pan, Yuanyuan Chen, Bo Liu, Na Li, Bo Tang

**Affiliations:** College of Chemistry, Chemical Engineering and Materials Science, Key Laboratory of Molecular and Nano Probes, Ministry of Education, Collaborative Innovation Center of Functionalized Probes for Chemical Imaging in Universities of Shandong, Institute of Molecular and Nano Science, Shandong Normal University Jinan 250014 P. R. China lina@sdnu.edu.cn tangb@sdnu.edu.cn

## Abstract

Breast cancer recurrence is the greatest contributor to patient death. As the immune system has a long-term immune memory effect, immunotherapy has great potential for preventing cancer recurrence. However, cancer immunotherapy is often limited due to T cell activation being blocked by insufficient tumor immunogenicity and the complex immunosuppressive tumor microenvironment. Here we show a tumor acidity activatable and Ca^2+^-assisted immuno-nanoagent to synergistically promote T cell activation and enhance cancer immunotherapy. When the immuno-nanoagent reaches the acidic tumor microenvironment, the CaCO_3_ matrix disintegrates to release immune stimulants (CpG ODNs and IDOi) and Ca^2+^. CpG ODNs are responsible for triggering dendritic cell maturation to increase the immunogenicity for activation of T cells. And IDOi can inhibit the oxidative catabolism of tryptophan to kynurenine for preventing T-cell anergy and apoptosis. Due to the complexity of the immunosuppressive microenvironment, it is difficult to restore T cell activation by inhibiting only one pathway. Fortunately, the released Ca^2+^ can promote the activation and proliferation of T cells with the support of the immune stimulants. *In vivo* experiments demonstrate that our Ca^2+^-assisted immuno-nanoagent can significantly suppress tumor progression and protect mice from tumor rechallenge due to the long-term memory effect. This immunotherapeutic strategy may provide more possibilities for clinical applications such as treating cancer and preventing relapse.

## Abstract 2

A tumor acidity activatable and Ca^2+^-assisted immuno-nanoagent was developed to enhance cancer immunotherapy and protect mice from tumor recurrence after surgery.

## Graphical abstract

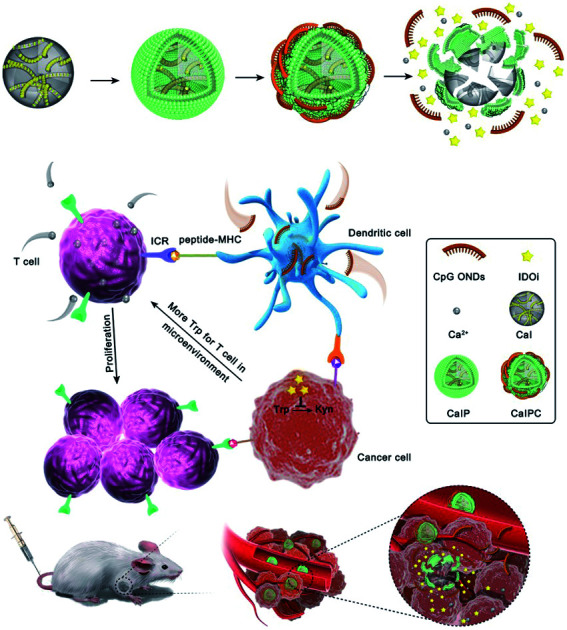

## Introduction

Breast cancer recurrence following the treatment of a primary tumor is the greatest contributor to patient death.^1,2^ As the direct cause of breast cancer is still unknown, it is hard to prevent its recurrence through interfering the key link during the treatment. Therefore, developing new strategies that are highly efficient in eradicating breast cancer and preventing its recurrence is the most urgent issue in clinics. Cancer immunotherapy, which harnesses the patients' own immune system to fight cancer, has made great strides in cancer treatment.^3–7^ In particular, the immune system has a long-term immune memory effect, which is beneficial for preventing cancer recurrence.^8,9^

Although checkpoint blockade immunotherapy is the most promising candidate of immunotherapy strategies for cancer therapy, it suffers from a low response rate due to blocked T cell activation by insufficient tumor immunogenicity and the complex immunosuppressive tumor microenvironment.^10^ Cytosine-phosphate-guanosine oligonucleotides (CpG ODNs), an immunostimulatory adjuvant, are a type of Toll-like receptor (TLR) agonist for triggering dendritic cells to enhance immunogenicity.^11–13^ However, this immune effect can be severely disrupted by the immunosuppressive tumor microenvironment.^14–19^ IDO, an immunoregulatory enzyme, that catalyses tryptophan (Trp) catabolism to produce kynurenine (Kyn), is highly overexpressed in tumor microenvironments.^20,21^ Increased Kyn with Trp depletion can inhibit T-cell proliferation and promote T-cell anergy and apoptosis, thus resulting in the suppression of antitumor immune responses.^22–25^ To block this suppression of antitumor immunity, the small-molecule IDO inhibitor INCB24360 (IDOi) was applied to inhibit the oxidative catabolism of Trp.^26^ In spite of the combination of CpG ODNs with immune checkpoint inhibitors showing amplified therapeutic benefits, immunotherapy is still ineffective in clinics, which is mainly due to the activation of T cells being blocked by various different immunosuppressive pathways. Therefore, it is of great significance to develop strategies that can directly activate T cells to strengthen the current immune regulators.

Emerging data suggest that calcium ions (Ca^2+^) are essential for T cell function and adaptive immunity.^27,28^ On the one hand, Ca^2+^ can specifically modulate the charge properties of lipids to provoke a key immune globulin, the T-cell receptor (TCR), for T-cell activation;^27^ on the other hand, inhibition of store-operated Ca^2+^ entry (SOCE), the main Ca^2+^ influx pathway in lymphocytes, will severely impair the antigen-dependent T cell proliferation.^28^ Therefore, it is promising to utilize Ca^2+^ to promote the activation and proliferation of T cells for regulating the immune response. Nevertheless, the application of Ca^2+^ in immunotherapy is limited in two aspects: (1) Ca^2+^, a kind of free metal ion, exhibits good solubility in water. Therefore, it is difficult to transport Ca^2+^ by the nanocarrier to the site of interest *in vivo* due to its leakage in physiological environments; (2) intravenous injection of Ca^2+^ easily leads to excessive Ca^2+^ in the blood, which will result in renal function damage, constipation, bone and joint pain, neurasthenia, *etc.* Therefore, it is of great significance to design a strategy that can generate Ca^2+^ in the site of interest to synergistically activate T cells and enhance immunotherapy.

**Fig. 1:**
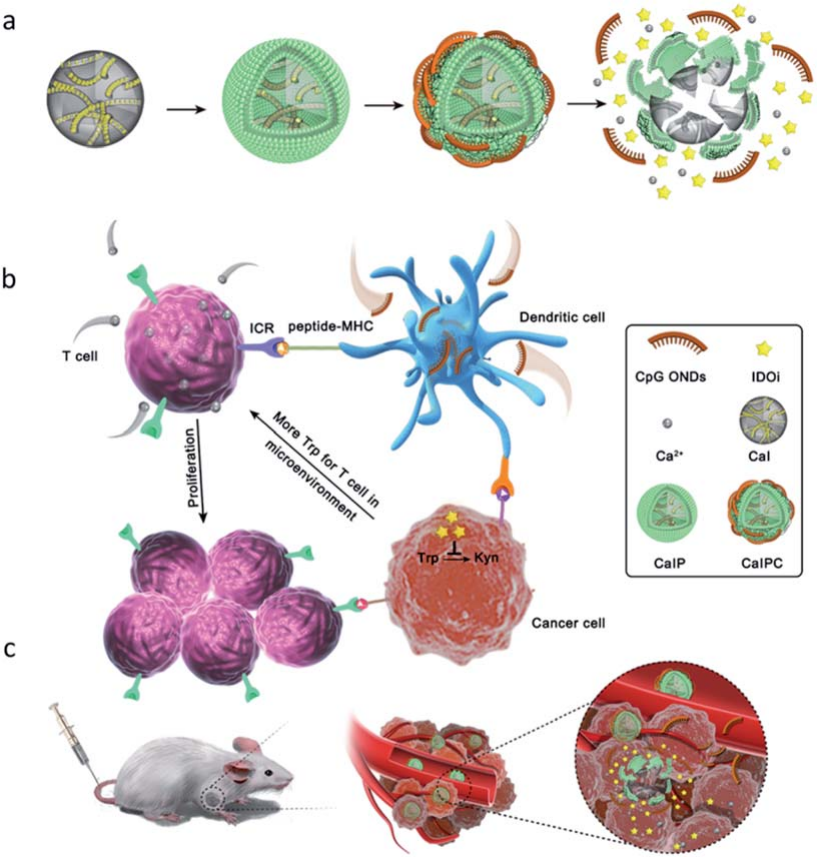
Schematic illustration of the tumor acidity activatable and Ca^2+^-assisted immuno-nanoagent (CaIPC) for enhancing breast cancer therapy and suppressing cancer recurrence. The preparation of the Ca^2+^-assisted immuno-nanoagent (a). The immune regulatory functions of the released immune stimulants (CpG ODNs and IDOi) and Ca^2+^ in detail (b). Intravenous injection of the Ca^2+^-assisted immuno-nanoagent into tumor-bearing mice for treating breast cancer and suppressing cancer recurrence (c).

Herein, we developed a tumor acidity activatable and Ca^2+^-assisted immuno-nanoagent that can generate Ca^2+^ in the tumor microenvironment for synergistically enhancing breast cancer therapy and suppressing cancer recurrence (Fig. 1). The immuno-nanoagent (CaIPC) is made of FDA-approved CaCO_3_ nanoparticles loaded with a small-molecule immunotherapy agent (IDOi) and functionalized with CpG ODNs. When injected intravenously, the CaCO_3_ matrix will specially disintegrate to release the CpG ODNs, IDOi and Ca^2+^ into the acidic tumor microenvironment. As the immunostimulatory adjuvants, CpG ODNs are responsible for promoting dendritic cell maturation to enhance immunogenicity. And IDOi is applied to inhibit the oxidative catabolism of Trp to Kyn for preventing T-cell anergy and apoptosis. Nevertheless, it is difficult to restore T cell activation by inhibiting only one pathway due to the complexity of the immunosuppressive microenvironment. Fortunately, the released Ca^2+^ can promote the activation and proliferation of T cells and synergizes with immune stimulants to ensure a strong and persistent autoimmune response. Our Ca^2+^-assisted immuno-nanoagent manifests superior performance in inhibiting tumor growth and defending against cancer recurrence due to the enhanced immune response and long-term memory effect. Therefore, this Ca^2+^-assisted immunotherapy strategy can provide new insights into future cancer immunotherapies in clinics.

## Results and discussion

### Preparation and characterization of CaCO_3_ nanoparticles

**Fig. 2:**
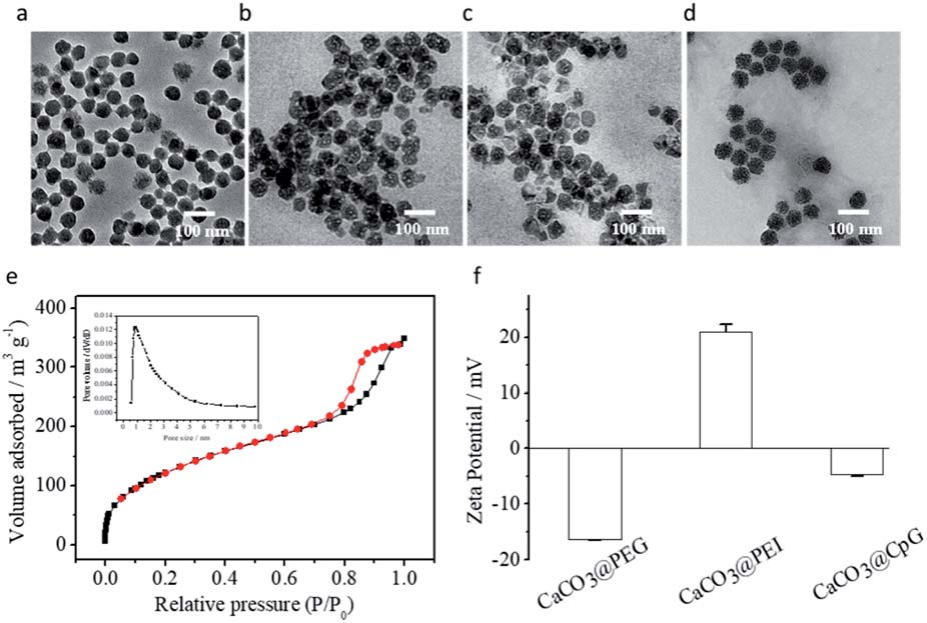
TEM images of CaCO_3_ nanoparticles in ethanol (a), CaCO_3_@IDOi@PEG in water (b), and CaCO_3_@IDOi@PEG@PEI in water (c), CaCO_3_@IDOi@PEG@PEI@CpG (CaIPC) in water (d). Scale bar = 100 nm. N_2_ adsorption–desorption isotherms of CaCO_3_ with an inset showing the pore size distribution obtained by the BJH method (e). Zeta potential of CaCO_3_@IDOi@PEG (CaCO_3_@PEG), CaCO_3_@IDOi@PEG@PEI (CaCO_3_@PEI), and CaCO_3_@IDOi@PEG@PEI@CpG (CaCO_3_@CpG) (f).

CaCO_3_ nanoparticles were prepared by the reported method with some modifications.^29^ Transmission electron microscopy (TEM) images showed that the CaCO_3_ nanoparticles were spherical and dispersed evenly in ethanol, with a uniform size of approximately 55–65 nm (Fig. 2a). The corresponding particle size distribution of CaCO_3_ nanoparticles was also 55–60 nm (Fig. S1a†). The N_2_ adsorption–desorption isotherms of CaCO_3_ nanoparticles are shown in Fig. 2e. The Brunauer–Emmett–Teller (BET) model was used to calculate the surface area values (200 m^2^ g^−1^). And the Barrett–Joyner–Halenda (BJH) model was applied to obtain the average pore diameters (∼1 nm), which indicated that the CaCO_3_ nanoparticles had a porous structure and drugs could be loaded inside pores. Next, a small-molecule immunotherapy agent, IDOi, was encapsulated into CaCO_3_ nanoparticles to form CaCO_3_@IDOi (CaI). The IDOi content was determined to be 12 μg mg^−1^*via*^19^F NMR spectroscopy (Fig. S2a and b†). Subsequently, CaI was modified with polyethylene glycol (PEG) and polyethylenimine (PEI) [CaCO_3_@IDOi@PEG and CaCO_3_@IDOi@PEG@PEI (abbreviated as CaIP)]. To quantify the amount of PEI coated on nanoparticles, thermogravimetric analysis (TGA) of CaCO_3_ with or without PEI was performed (Fig. S3†). The amount of PEI coated on the nanoparticles was calculated to be 70.3 μg mg^−1^ CaCO_3_. As shown in Fig. 2b and c, the as-synthesized CaCO_3_@IDOi@PEG and CaIP nanoparticles dispersed evenly with an average size of 55–65 nm. The particle size distributions also showed that the nanoparticles had an average diameter of 55–65 nm (Fig. S1b and c†). Subsequently, CaIP nanoparticles were modified with immunostimulatory CpG ODNs through an electrostatic interaction between the amino group of PEI and the phosphate backbone of the CpG ODNs to form the immuno-nanoagent CaCO_3_@IDOi@PEG@PEI@CpG (abbreviated as CaIPC). The nanodrop assay was performed to show that the content of CpG ODNs was 10 μg mg^−1^ CaCO_3_. In addition, CpG ODNs were modified with carboxyfluorescein (CpG ODN-FAM) to further verify the CpG ODN content (10 μg mg^−1^) by fluorescence quantitative analysis. The fluorescence spectra and standard curve of CpG ODN-FAM are shown in Fig. S2c and d.† Then release profiles of Ca^2+^ and IDOi were studied. It is shown that Ca^2+^ and IDOi are released faster at pH 6.5 than at pH 7.4 from the start. The release rates slow down at 2 h and become gentle. The release amount of Ca^2+^ is ∼60% at pH 6.5 and ∼20% at pH 7.4 due to the greater solubility in acid solution (Fig. S4a†). And for IDOi, it is >60% at pH 6.5 and >30% at pH 7.4 within 4 h (Fig. S4b†). In addition, the CpG ODNs were also released faster at pH 6.5 than at pH 7.4. The release amount of CpG ODNs is ∼60% at pH 6.5, while ∼40% at pH 7.4 within 4 h (Fig. S5†). The CaIPC nanoparticles had a good morphology with a size of 55–65 nm (Fig. 2d and S1d†). The dynamic light scattering (DLS) data showed that the hydrodynamic size of CaIPC in water was approximately 120 nm in aqueous solution (Fig. S6a†). For comparison, a control immuno-nanoagent with a liposome as the nanocarrier was prepared (LIPC), and the hydrodynamic size of LIPC was approximately 100 nm (Fig. S6b†). Zeta potential experiments depicted in Fig. 2f illustrated the successful fabrication of CaIPC, *i.e.*, −16.4 ± 0.2 mV (CaCO_3_@IDOi@PEG), +21.0 ± 1.4 mV (CaCO_3_@IDOi@PEG@PEI) and −4.7 ± 0.4 mV (CaCO_3_@IDOi@PEG@PEI@CpG).

### Dissolution of CaCO_3_ nanoparticles under acidic conditions

**Fig. 3:**
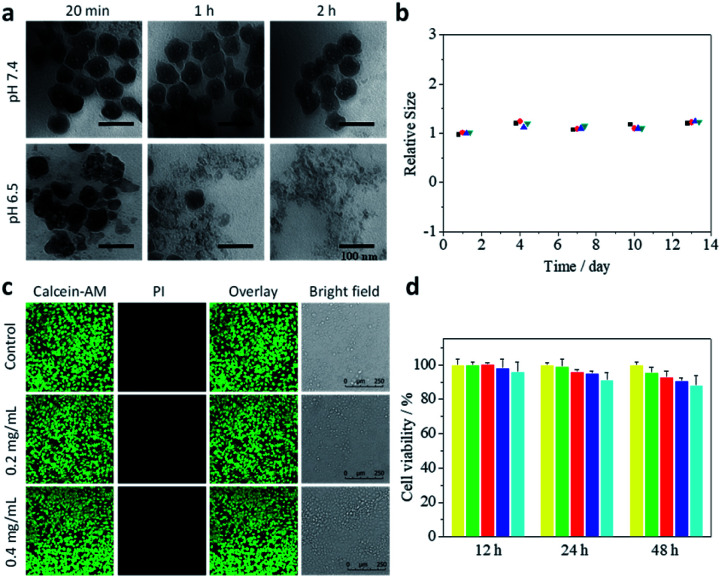
TEM images of CaIPC in buffers with different pH values (7.4 and 6.5). Scale bar = 100 nm (a). Stability of CaIPC in buffer (pH 7.4) by DLS (*n* = 4) (b). Cell viability as determined in a live/dead assay (c). MTT assay of 4T1-Luc cells incubated with 0, 0.05, 0.1, 0.2 or 0.4 mg mL^−1^ CaIPC for 12, 24 and 48 h (d).

Next, the release of Ca^2+^ from CaIPC nanoparticles into an acidic microenvironment was investigated. CaIPC nanoparticles were immersed in various buffers (6.5 and 7.4) to test their pH sensitivity. TEM images at different time points showed that CaIPC nanoparticles were quite stable at pH 7.4 and showed no notable changes of size or structure (Fig. 3a). In contrast, these nanoparticles gradually disintegrated and mostly lost their spherical morphology after 2 h at pH 6.5. It indicated that the acid-triggered CaCO_3_ should be advantageous for long existence in blood circulation at physiological pH and quick disintegration in the tumor microenvironment at low pH. To further verify the stability, CaIPC particles were immersed in buffer (pH 7.4), and their hydrodynamic sizes were measured for 13 days by DLS. Fig. 3b shows that the hydrodynamic size was constant for 13 days and had good repeatability, which indicated that the CaIPC nanoagent presented good stability and was propitious to circulate *in vivo*.

### Biocompatibility of CaIPC

To evaluate the biocompatibility of CaIPC, we studied cytotoxicity by means of an *in vitro* MTT assay. 4T1-Luc cells in 96-well plates were incubated with 0, 0.05, 0.1, 0.2 or 0.4 mg mL^−1^ CaIPC for 12, 24 and 48 h. The viability of all cells was greater than 88%, demonstrating that CaIPC nanoparticles had low cytotoxicity (Fig. 3d). The values in this study were expressed as mean ± SD and had good repeatability. The corresponding bioluminescence images of 4T1-Luc cells under the same conditions as mentioned above demonstrated that the cells maintained good vitality (Fig. S7†). Then, cell viability was further determined using calcein-acetoxymethyl ester (calcein-AM) and propidium iodide (PI) in a live/dead assay. Calcein-AM is cleaved by esterases within live cells to fluoresce green, while PI can permeate only dead membranes and fluoresces red by binding to nucleic acids. As shown in the confocal images (Fig. 3c), 4T1-Luc cells incubated with 0.2 mg mL^−1^ and 0.4 mg mL^−1^ CaIPC for 48 h fluoresced green, indicating that the synthesized CaIPC presented good biocompatibility and is therefore suited for intravenous injection *in vivo*.

### Immune activation

**Fig. 4:**
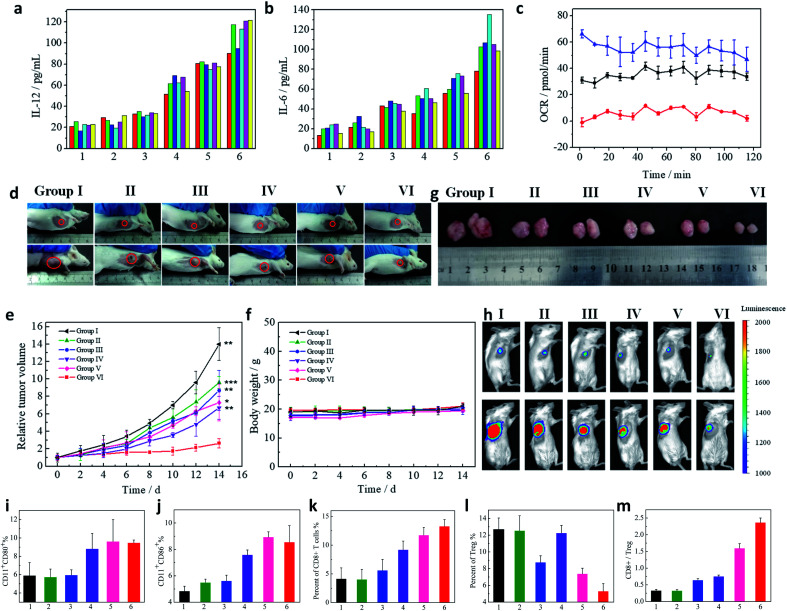
Serum IL-12 (a) and IL-6 (b) levels of mice treated for 12 h with 1, NS; 2, CaP; 3, CaIP; 4, CaPC; 5, LIPC; and 6, CaIPC. The colorful columns represent six mice in each group. The mitochondrial oxygen consumption rate (OCR) of lymphocytes treated with RPMI 1640 (Black), RPMI 1640 with Ca^2+^ (Blue), or RPMI 1640 with BAPTA-AM (Red) (c). *In vivo* tumor therapy and antitumor efficacy. Photographs of mice taken before treatment (day 0) and at day 14 with different treatments: I, NS; II, CaP; III, CaIP; IV, CaPC; V, LIPC; and VI, CaIPC (d), tumor growth curves (e) and mice body weight curves (f) (*n* = 8). *P* values were calculated using the *t*-test (***P < 0.001, **P < 0.01, and *P < 0.05) to compare other groups with group VI. The data are presented as mean ± SD. Photographs of tumors harvested from the mice after 14 days (g). Bioluminescence images were obtained before treatment (day 0) and at day 14 with different treatments (h). Flow cytometry analysis of the CD 80 (i) and CD 86 (j) expressions of splenocytes. Proportions of CD8+ T cells (gated on CD3+ T cells) (k), Tregs (l), and CD8+/Tregs (m) in tumors of mice treated with I, NS; II, CaP; III, CaIP; IV, CaPC; V, LIPC; and VI, CaIPC. The injected doses of IDOi and CpG ODNs were respectively 600 μg kg^−1^ and 500 μg kg^−1^ each time.

To assess therapeutic potential of CaIPC, *in vivo* immunostimulatory activity was investigated. Cytokines, such as IL-6 and IL-12, produced by immune cells are essential regulators in modulating immune responses.^30^ Therefore, the levels of secreted cytokines were measured to assess immune activation. Tumor-bearing mice were administered the following *via* intravenous injection: I, normal saline (NS); II, CaCO_3_@PEG@PEI (CaP); III, CaCO_3_@IDOi@PEG@PEI (CaIP); IV, CaCO_3_@PEG@PEI@CpG (CaPC); V, liposome@IDOi@PEG@PEI@CpG (LIPC); and VI, CaCO_3_@IDOi@PEG@PEI@CpG (CaIPC) (50 mg kg^−1^). After 12 h, the mice were sacrificed to harvest blood serum for measuring the levels of secreted cytokines by enzyme-linked immunosorbent assay (ELISA). The six treatment groups displayed different extents of induced immune responses, as reflected by cytokine expression levels (Fig. 4a and b). It was found that the mice in groups I and II showed low serum levels of IL-6 and IL-12, indicating that the CaP nanoparticles had negligible immunostimulatory activity. The serum levels of secreted IL-12 and IL-6 were obviously increased in group IV, demonstrating that CpG ODNs have significant immunostimulatory activity. However, group III with CaIP exhibited only a slight induction of IL-6 and IL-12, demonstrating that IDOi alone cannot effectively trigger an immune response. Nonetheless, groups V and VI dramatically induced the high production of serum IL-6 and IL-12, indicating that CpG ODNs and IDOi cooperated with each other and boosted the antitumor immune response (Fig. 4a and b). Significantly, the levels of cytokines in group VI were higher than those in group V, which suggested that CaCO_3_ played a vital role during the treatment. Interferon γ (IFN-γ) is secreted by activated T cells and natural killer (NK) cells, which has immunomodulatory and anti-tumor properties. Tumor necrosisfactor-α (TNF-α) is mainly produced by macrophages, which can directly cause the death of tumor cells. Moreover, IFN-γ synergizes with TNF-α to play a vital role in cancer immunotherapy. Therefore, the secretions of IFN-γ and TNF-α of mice with different treatments were also measured by ELISA. It was shown that the levels of IFN-γ and TNF-α increased mainly in the order from group I to VI (Fig. S8†). Their levels in group VI were the highest, indicating that our nanoagent can effectively regulate the body's immune response. To further evaluate the inherent immune-stimulatory activity of our nanoagent, the activation of dendritic cells was studied. Groups IV, V and VI showed higher expression levels of CD80 and CD86 in dendritic cells, suggesting that nanoparticles with CpG ONDs can efficiently activate the maturation of dendritic cells to serve as antigen-presenting cells (APCs) (Fig. 4i and j). The maturation of APCs further indicated the activation immunity by the nanoagent.^8,31^ As is well-known, CD8+ T cells could directly kill the targeted tumor cells.^13^ So CD8+ T cells in the tumors were also studied after treatment with various nanoagents to verify the activation immunity. Flow cytometry data in Fig. 4k showed that the percentages of CD8+ T cells (gated on CD3+ T cells) in groups I to VI were ∼4.16%, 4.05%, 5.61%, 9.20%, 11.74% and 13.33%. Regulatory T cells (Tregs) are important cells for mediating immunoregulation to maintain self-tolerance. Tregs (CD3+CD4+Foxp3+) significantly decrease in groups III, V and VI, which suggests that IDOi could greatly reduce the percentages of Tregs in tumors to relieve immunosuppression (Fig. 4l). The ratio of CD8+/Tregs is the greatest in group VI, indicating that the cell immunity is significantly enhanced for cancer immunotherapy (Fig. 4m). The result suggests that our nanoagent with CaCO_3_, IDOi, and CpG has higher immunostimulatory activity than all other groups, which is in accordance with the ELISA data in Fig. 4 and S8.†

CpG ODNs have been commonly utilized as immune modulators to stimulate an immune response.^31,32^ So groups treated with CpG ODNs (groups IV, V and VI) clearly evidenced the activation of dendritic cells (Fig. 4i and j) and the secretion of IL-6 and IL-12 compared to group I (Fig. 4a and b). Nevertheless, a majority of cancers fail to respond to immunotherapy due to immunosuppression, which can prevent immune cells from attacking cancer cells.^33,34^ To block the suppression of antitumor immunity, the small-molecule IDO inhibitor (IDOi) was applied in this work. To exploit the IDO inhibitory effect of our material CaIPC, Kyn and Trp were detected by liquid chromatography (LC). 4T1 cells were seeded in culture dishes, and IFN-γ (100 ng mL^−1^) was added to each dish to stimulate the IDO expression. CaIPC was pre-incubated in pH 6.5 buffer for 4 h and then added to the cells. After 24 h, the cell culture medium of each dish was collected and trichloroacetic acid was added to precipitate the proteins. After centrifugation, the supernatant was collected for the analysis of Kyn and Trp using LC. As shown in Fig. S9,† Kyn/Trp of the cells treated with IFN-γ increased obviously, indicating that IFN-γ can upregulate the IDO expression in cancer cells to catalyze Trp into Kyn. Cells treated with IFN-γ and our material (IFN + M) showed the low level of Kyn/Trp, the same as the control, which indicated the IDO inhibitory effect of our material *in vitro*. The IDO inhibitory effect of our materials was further studied *in vivo*. Tumor-bearing mice were administered the following *via* intravenous injection: 1, NS; 2, CaP; 3, CaIP; 4, CaPC; 5, LIPC; and 6, CaIPC. After treatment, tumors were harvested to obtain the homogenate using a homogenizer. Then the homogenate was dissolved in 10% trichloroacetic acid to precipitate the proteins. Kyn and Trp in the supernatant were then examined using LC. It was observed that materials with IDOi (groups 3, CaIP; 5, LIPC; and 6, CaIPC) presented low levels of Kyn/Trp, indicating their IDO inhibitory effect (Fig. S10†). However, IDOi itself cannot trigger immune responses and plays the role of assisting CpG ODNs. Therefore, group III with only IDOi, exhibited no activation of dendritic cells, only merely a slight induction of the IL-6 and IL-12 expression, and a slight percentage of CD8+ T cells (Fig. 4). In summary, CpG ODNs are responsible for effectively stimulating an immune response, and IDOi for enabling the immune response to proceed efficiently.

More importantly, recent studies have found that Ca^2+^ plays a vitally important role in immunoregulation by controlling T-cell receptor activation and the Ca^2+^ influx pathway.^27,28^ Analysing the metabolic activity of lymphocytes with and without Ca^2+^ is a straightforward approach to study the role of Ca^2+^ in immune responses. The oxygen consumption rate (OCR) is an important indicator of cell metabolism and function.^35,36^ And a high OCR indicates a high metabolic rate and good cell viability. To analyse the impact of Ca^2+^ on lymphocytes, mitochondrial OCRs of lymphocytes were studied. Fig. 4c shows that the OCR of lymphocytes in an RPMI 1640 medium was ∼30 pmol min^−1^. When Ca^2+^ was added, the OCR rose to approximately 55 pmol min^−1^. In addition, when Ca^2+^ was chelated with BAPTA-AM, the OCR decreased to 0–5 pmol min^−1^, demonstrating that Ca^2+^ plays a vital role in lymphocyte viability. All values in the present study were expressed as mean ± SD. And the data showed that the experiments had good repeatability. Fig. 4a and b also show that IL-6 and IL-12 expression levels in group VI are higher than those in group V, which further demonstrates that Ca^2+^ plays a vitally important role in immune responses.

### Tumor immunotherapy model

Antitumor immunotherapy effects of CaIPC in a murine tumor model were further explored. For comparison, tumor-bearing mice were treated with the following: I, NS; II, CaP; III, CaIP; IV, CaPC; V, LIPC; and VI, CaIPC (50 mg kg^−1^) every other day for 5 treatments. Changes in the tumor volumes were monitored over a period of 14 days. The CaIPC-treated mice in group VI showed the slowest rate of tumor progression, indicating that CaIPC effectively inhibited tumor growth. In contrast, groups II, III, IV, and V showed moderate tumor suppression compared with group I (Fig. 4d, e and g). This indicated that group VI with CpG ODNs, IDOi, and Ca^2+^ had a commendable immunotherapy effect. However, the absence of any one of these factors reduced the antitumor effect. Tumor sizes were also monitored with bioluminescence signals of 4T1-Luc cells at day 0 and day 14 (Fig. 4h), and the corresponding bioluminescence quantification was performed (Fig. S11†). The bioluminescence images of the mice showed that the CaIPC-treated mice had the smallest tumor volumes, which is consistent with the data in Fig. 4d, e and g and S11.†

To further verify the treatment effect of the nanoagent, the therapeutic trials in an orthotopic mouse tumor model were conducted. Firstly, 10^5^ 4 T1 cells were inoculated in the mice mammary fat pads. 5 days later, the mice were treated with nanoagents (50 mg kg^−1^) every other day for 5 treatments. After 15 days, orthotopic 4T1 tumors were harvested from mice. As can be seen from Fig. S12,† the tumors of mice treated with CaIPC in group VI was the smallest, indicating that CaIPC can effectively inhibit tumor growth in the 4T1 orthotopic model. In contrast, groups II, III, IV, and V showed moderate tumor suppression compared with group I. It indicated that our nanoagent led to significant tumor suppression in the orthotopic murine model.

Body weight is an important parameter for evaluating the systemic toxicity of a material to the body. As shown in Fig. 4f, the body weights of all the groups did not obviously change for 14 days, implying that the treatments had no unpleasant side effects. Moreover, haematoxylin and eosin (H&E) staining of five major organs (the heart, liver, spleen, lungs and kidneys) was carried out at 12 h and 24 h post-treatment. No histopathological abnormalities were found in any group, proving that the immune response provoked by nanoparticles led to few side effects (Fig. S13†). In further experiments, CaCO_3_ nanoparticles doped with Mn (CaCO_3_@Mn) were synthesized according to the published article^29^ for analyzing the distribution of the nanoagent by detecting the Mn element *via* ICP-AES. Then the nanoagent was prepared with CaCO_3_@Mn instead of CaCO_3_. Mice with tumors were injected intravenously with the nanoagent. At 24 h post-injection, the mice were sacrificed and five major organs were dissolved in *aqua regia* (HCl : HNO_3_ : HClO_4_ = 3 : 1 : 2, v/v/v) for analysis of Mn. The result indicated that the nanoagent was accumulated in tumors with about 7.4 ID%/g by the enhanced permeability and retention effect (Fig. S14†). Fig. S15† shows that the nanoagent was mainly distributed in the liver and kidneys. Blood biochemical tests further showed that alanine transaminase (ALT), aspartate aminotransferase (AST), blood urea nitrogen (BUN) and creatinine (CR) were all in the normal range, indicating that the nanoagent had no influence on the liver and kidneys (Fig. S16†). Blood routine indices such as the amounts of red blood cells, platelets *etc.* were all in a normal level demonstrating that the nanoagent had no influence on normal physiological activity. Besides, the amounts of lymphocytes in blood routine tests were higher in the experimental group than those in control groups, which was due to the activation of the immune response by our nanoagent (Fig. S17†). Furthermore, mice with tumors were injected intravenously with the nanoagent. Then the blood was harvested and dissolved in *aqua regia* for measurements of Mn at different time points. It is shown that Mn signals in blood samples declined over time following a two-compartment model, with the first (*t*_1/2_(a)) and second (*t*_1/2_(b)) phases of circulation half-lives determined to be 0.95 ± 0.54 and 13.15 ± 0.87 h, respectively (Fig. S18†). After injected intravenously with the nanoagent, the urine and feces of mice at various time points were collected and dissolved in *aqua regia* for analysis of Mn. The results indicated that the nanoagent was metabolized through urine and feces with time (Fig. S19†). All values in the present studies were expressed as mean ± SD. And the results showed that the experiments had good repeatability. These data demonstrated that CaIPC immunotherapy had good biocompatibility and led to significant tumor suppression in murine models, which reveals the great potential of this nanoagent in cancer immunotherapy applications.

### Long-term immune memory effects

**Fig. 5:**
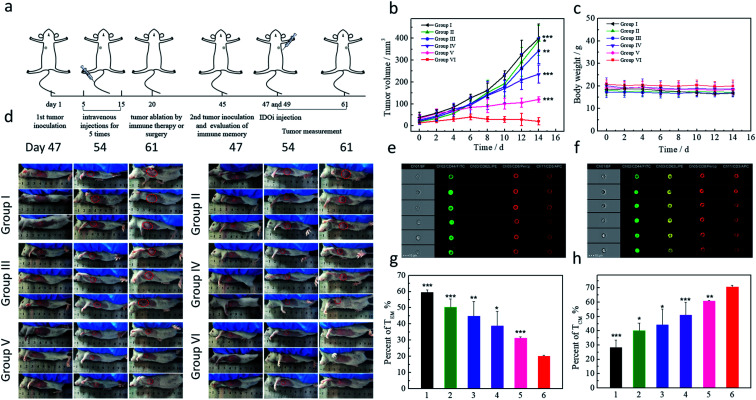
Long-term immune memory effects. Schematic illustration of the experimental process (a). Tumor growth curves of tumors present after a rechallenge by reinoculation at day 45 post-elimination of the first tumors (b) and body weight curves (c) with different treatments at day 5 with 15: I, NS; II, CaP; III, CaIP; IV, CaPC; V, LIPC; and VI, CaIPC (*n* = 8). The injected doses of IDOi and CpG ODNs were respectively 600 μg kg^−1^ and 500 μg kg^−1^ each time. Photographs of the mice at days 47, 54 and 61 (d). Analysis of memory T cells by flow cytometry (gated on CD3+CD8+ T cells): effector memory T cells (T_EM_) (e) and central memory T cells (T_CM_) (f) in the lymph nodes at day 45; proportions of T_EM_ (g) and T_CM_ (h). *P* values were calculated using the *t*-test (***P < 0.001, **P < 0.01, and *P < 0.05) to compare other groups with group VI. The data are presented as the mean ± SD.

The immune system has the capacity to remember pathogens for years, which is vitally important for preventing disease recurrence. Therefore, it was necessary to investigate the long-term immune memory effects induced by CaIPC. Mice were inoculated with 4T1 tumors in the left flank and treated every other day for 5 treatments with the following: I, NS; II, CaP; III, CaIP; IV, CaPC; V, LIPC; and VI, CaIPC (50 mg kg^−1^). Then, all tumors were eliminated by immunotherapy or surgery at day 20. For the second tumor inoculation, 4T1-Luc cells were subcutaneously injected into the right flank of the mice at day 45, and the mice were then injected with IDOi at day 47 and day 49 (1 mg kg^−1^) (Fig. 5a). The body weights and tumor sizes were measured for another 14 days and all values in the present studies were expressed as mean ± SD (Fig. 5). The results substantiated that tumors in the group V LIPC-treated mice were relatively slow-growing (Fig. 5b and d), suggesting that LIPC induced long-term immune memory to some degree. In addition, the re-inoculated tumors were obviously inhibited by the CaIPC treatment in group VI, and approximately 40% of the tumors completely disappeared, suggesting good long-term immune memory effects of CaIPC (Fig. 5b and d). Furthermore, tumors in group IV, with the CaPC treatment, showed only a small degree of tumor inhibition due to the unsatisfactory immune activity induced by only Ca^2+^ and CpG ODNs. Hence, group IV appeared to be incompetent in the immune memory effect required to protect mice from a tumor rechallenge. 8 mice of each group were studied and the experiment data showed good repeatability. Notably, the bioluminescence images also showed that group VI had the weakest luciferase signal, suggesting the presence of minimal tumors, which is consistent with the above results (Fig. S20†). All these experimental results indicated that group VI, with CpG ODNs, IDOi, and Ca^2+^, exhibited good long-term memory immune effects that could trigger an immune response again during the tumor rechallenge and thereby prevent tumor recurrence in these mice.

To further confirm the long-term immune memory effects of CaIPC, flow cytometry assays were conducted. Based on their function, proliferation, and migration, memory T cells were divided into two categories: central memory T cells (T_CM_) and effector memory T cells (T_EM_). Moreover, T_CM_ and T_EM_ were defined as CD3+CD8+CD62L+CD44+ and CD3+CD8+CD62L−CD44+, respectively (Fig. 5e and f). It has been reported that the long-term persistence of memory T cells is primarily in the form of T_CM_.^37,38^ Therefore, the proportions of T_CM_ and T_EM_ before the second tumor inoculation were measured. The results showed that the Balb/c mice treated with CaIPC had a higher proportion of T_CM_ in the lymph nodes (VI, ∼71%) than did the other groups (I, ∼28%, II, ∼40%, III, ∼44%, IV, ∼51%, and V, ∼61%) (Fig. 5g and h). The values in the present study were expressed as mean ± SD and the experiments had good repeatability. Overall, these results revealed that CaIPC nanoagents had more powerful immune memory effects than the other groups.

### Prevention of tumor recurrence

**Fig. 6:**
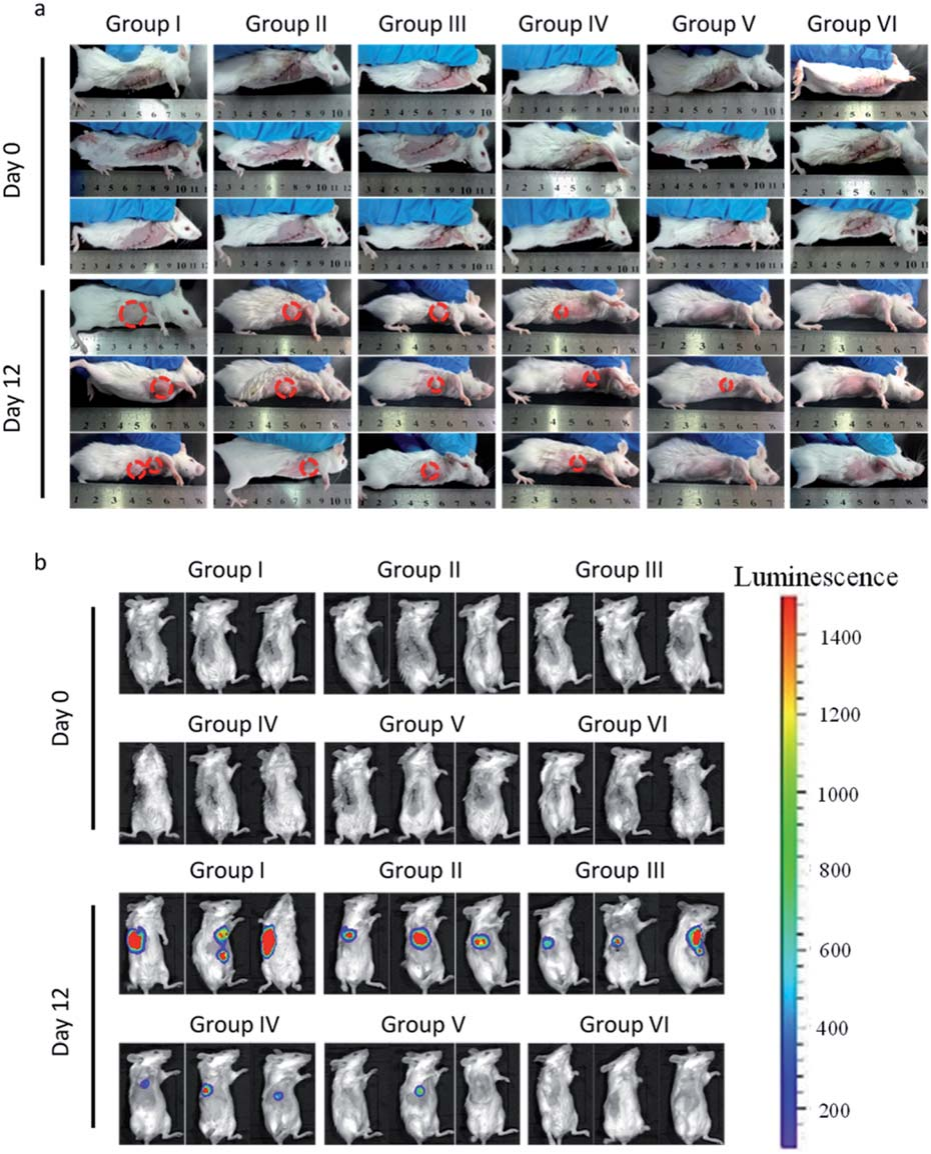
*In vivo* tumor immunotherapy to reduce post-surgical tumor relapse by CaIPC. Photographs of mice (a) and *in vivo* bioluminescence images of the 4T1-Luc tumors (b) of different groups after the removal of the primary tumor (group I, NS; II, CaP; III, CaIP; IV, CaPC; V, LIPC; and VI, CaIPC), and various treatments are indicated. The injected doses of IDOi and CpG ODNs were respectively 600 μg kg^−1^ and 500 μg kg^−1^ each time.

The ability of CaIPC to prevent tumor recurrence following resection was further evaluated. Tumor-bearing mice were randomly divided into six groups (*n* = 8) after tumors were incompletely resected, leaving behind ∼1% residual tissue, and were subjected to different treatments: I, NS; II, CaP; III, CaIP; IV, CaPC; V, LIPC; and VI, CaIPC (50 mg kg^−1^) every other day for 5 treatments. All tumors recurred following resection when mice were treated with NS, suggesting that breast cancer can easily relapse after surgery. Tumors treated with CaP, CaIP, or CaPC post-resection all recurred to different degrees, suggesting that the lack of either CpG ODNs or IDOi will attenuate the immunotherapy effect. However, only ∼30% tumors recurred following resection when mice were treated with LIPC, suggesting that CpG ODNs and IDOi can synergistically trigger the immune response to kill cancer cells. Moreover, CaIPC treatment post-resection can completely destroy the remaining cancer cells, thus preventing tumor re-growth (Fig. 6a). In summary, CpG ODNs, IDOi, and Ca^2+^ had a synergistic immunotherapy effect, and the absence of any one of these factors interfered with this therapeutic effect. Bioluminescence images also showed that tumors in groups I–IV all recurred to different degrees, and ∼30% of tumors recurred in group V, whereas none of the tumors in group VI relapsed (Fig. 6b). The quantification of the corresponding bioluminescent tumors of the different groups of mice after various treatments also indicated the capacity of CaIPC to prevent tumor relapse (Fig. S21†). In addition, the capacity of CaIPC to prevent cancer recurrence by intravenous or peritumoral injection was verified. After incomplete tumor resection that left behind ∼1% residual tissue, both intravenous and peritumoral injection of CaIPC had the ability to prevent cancer recurrence, indicating that the intravenous injection of CaIPC showed the same performance as peritumoral injection in eliciting an immune response to attack any minimal residual tumor (Fig. S22†). All these results verified that our immuno-nanoagent has the potential to prevent cancer recurrence.

## Conclusions

In summary, we have presented a tumor acidity activatable and Ca^2+^-assisted immuno-nanoagent for simultaneously activating T cells to enhance immunotherapy. In this system, the immuno-nanoagent is made of FDA-approved CaCO_3_ nanoparticles loaded with IDOi and functionalized with CpG ODNs. The CaCO_3_ matrix would typically disintegrate in the acidic tumor microenvironment to release CpG ODNs, IDOi and Ca^2+^ to synergistically activate T cells; (1) CpG ODNs as immunostimulatory adjuvants can promote dendritic cell maturation and then present the antigen to the T cells; (2) IDOi is applied to inhibit the oxidative catabolism of Trp to Kyn for preventing T-cell anergy and apoptosis; (3) the released Ca^2+^ can modulate the charge properties of lipids and maintain the main Ca^2+^ influx pathway to promote the activation and proliferation of T cells. Compared with previously reported strategies, the clinical safety of the current approach benefits from the fact that the immune response is provoked specifically within the acidic tumor microenvironment. Significantly, the released Ca^2+^ can promote the activation and proliferation of T cells against the complex immunosuppressive microenvironment. Moreover, compared with monomeric immune-nanoagents, the tumor inhibition rate of our multicomponent synergistic immune-nanoagent is above 90%, which indicates the better therapeutic effect against breast cancer in mouse models. In addition, secondary tumors inoculated after immunotherapy or surgery exhibited significant growth inhibition or even ablation due to the enhanced long-term immune memory effect of the immune system. Tumor recurrence assays further verified that our Ca^2+^-assisted immuno-nanoagent has the capacity to prevent tumor relapse effectively. We anticipate that this immuno-nanoagent will not only have potential application for breast cancer treatment in clinics, but also pave the way for providing further effective strategies for immunotherapy of other tumor types.

## Ethical statement

All the animal experiments were conducted in agreement with the Principles of Laboratory Animal Care (the People's Republic of China) and the Guidelines of the Animal Investigation Committee, Biology Institute of Shandong Academy of Science, China.

## Conflicts of interest

The authors declare no competing financial interest.

## Supplementary Material

SC-011-D0SC00293C-s001
