# Supplementary material for: A tumor acidity activatable and Ca2+-assisted immuno-nanoagent enhances breast cancer therapy and suppresses cancer recurrence
Source: Chem Sci. 2020 Jun 29;11(28):7429–37. doi: 10.1039/d0sc00293c (PMC8159290; doi:10.1039/d0sc00293c)
Supplement: SC-011-D0SC00293C-s001 [file SC-011-D0SC00293C-s001.pdf]

Electronic Supplementary Material (ESI) for Chemical Science.
This journal is © The Royal Society of Chemistry 2020

Supporting Information

A tumor acidity activatable and Ca^{2+}-assisted immuno-nanoagent enhances breast cancer therapy and suppresses cancer recurrence

Yanhua Li, Shaohua Gong, Wei Pan, Yuanyuan Chen, Bo Liu, Na Li*, and Bo Tang*

College of Chemistry, Chemical Engineering and Materials Science Key Laboratory of Molecular and Nano Probes, Ministry of Education Collaborative Innovation Center of Functionalized Probes for Chemical Imaging in Universities of Shandong, Institute of Molecular and Nano Science, Shandong Normal University Jinan, 250014 (P. R. China).

Reagents and Materials.

Calcium chloride dihydrate ($CaCl_2 \cdot 2H_2O$) and ammonia bicarbonate (NH_4HCO_3) were purchased from China National Pharmaceutical Group Corporation, China. Polyethylenimine (PEI, M.V. 10000) was purchased from Macklin, China. 1, 2-dioleoyl-sn-glycero-3-phosphate (sodium salt) (DOPA) and 1,2-distearoyl-sn-glycero-3-phosphoethanolamine-N-(methoxy(polyethylene glycol)-2000) DSPE-PEG2000 were purchased from Avanti. 1, 2-dihexadecanoyl-sn-glycero-3-phosphocholine (DPPC) was purchased from Corden Pharma Switzerland LLC. Cholesterol was purchased from Xi'an ruixi Biological Technology Co., Ltd. The INCB24360 analogue, 4-amino-N-(3-chloro-4-fluorophenyl)-N'-hydroxy-1,2,5-oxadiazole-3-carboximidamide (IDO5L) (IDOi) was purchased from Medchemexpress (MCE). Cytosine-phosphate-guanine oligonucleotides (CpG ODNs): 5'-TCCATGACGTTCCTGACGTT-3' were purchased from Sangon Biotech Co., Ltd. Fetal bovine serum (FBS) was purchased from Biological Industries. Trypsin-EDTA was purchased from Gibco, USA. RPMI 1640 medium was purchased from Key Gen Biotech. Co., Ltd. The Mouse IL-12 Mini ELISA Kit and Mouse IL-6 ELISA Kit were purchased from Elabscience Biotechnology Co., Ltd. The Mouse INF-γ Mini ELISA Kit and Mouse TNF-α ELISA Kit were purchased from Boster Biological Technology Co., Ltd, China. Flow cytometric antibodies were purchased from BioLegend, Inc. D-luciferin was purchased from EFEBIO Co., Ltd. 4T1-Luc cells were purchased from Shanghai AOLU Biological Technology Co., Ltd. Puromycin was purchased from Beijing Solarbio Science & Technology Co., Ltd. Glass Bottom dishes were purchased from Cellvis, Mountain View, CA. 96-well plates were purchased from Hangzhou Xinyou Biotechnology Co., Ltd, China. Plastic centrifuge tubes were purchased from GeneBrick Bioscience LLC. Red blood cell lysis buffer was purchased from Sangon Biotech Co., Ltd. All the aqueous solutions used in experiments were prepared using deionized water (18.2 MΩ cm) obtained from a Milli-Q water purification system. All chemicals were of analytical grade and were used without further purification. Transmission electron microscopy (TEM) was carried out on a HT7700 electron microscope (Hitachi, Japan). The *in vivo* imaging study was performed with a Caliper IVIS Lumina III imaging system (Caliper Co., USA).

The flow cytometer assays were carried out with an imaging flow cytometer (Amnis Corporation, USA), and IDEAS image analysis software (Amnis) was used to analyze the images. The optical density (OD value) of was measured with an enzyme labelling apparatus (Thermo scientific, USA). Oxygen consumption rate (OCR) was performed with Extracellular Flux Analyzer (Seahorse bioscience, USA).

Synthesis of the immuno-nanoagent $CaCO_3$@IDOi@PEG@PEI@CpG (CaIPC) nanoparticles.

$CaCO_3$ nanoparticles were synthesized by means of a gas diffusion reaction. Briefly, 220 mg $CaCl_2 \cdot H_2O$ was dissolved in 100 mL ethanol in a glass bottle covered with aluminium foil, which was punctured with several pores. Then, the bottle was put into a vacuum drying chamber containing 8 g dry ammonia bicarbonate (NH_4HCO_3). After keeping the whole system in a vacuum environment for 24 h, $CaCO_3$ nanoparticles were obtained and were separated by centrifugation at 12000 rpm. Then, 2 mg $CaCO_3$ was mixed with IDOi in DMSO (2 mg/mL) to obtain $CaCO_3$@IDOi (abbreviated as CaI). The mixture was stirred for at least 24 h to attain maximum loading.

A two-step approach was conducted to modify the above nanoparticles with PEG and PEI. First, 20 mg CaI in ethanol solution and 1 mL DOPA solution (2 mg/mL in chloroform) were mixed under ultrasonication for 20 min. The obtained turbid solution was centrifuged to remove unbound free DOPA and redispersed in chloroform. PEGylation of the nanoparticles was then conducted by mixing a chloroform solution of DPPC, cholesterol and DSPE-PEG at a 4:4:2 M ratio with $CaCO_3$-DOPA under vigorous stirring overnight. Afterwards, the chloroform was evaporated, and the obtained $CaCO_3$@IDOi@PEG nanoparticles were dissolved in aqueous solution for further use. Then, the obtained nanoparticles were stirred with 200 mg PEI for 24 h to prepare $CaCO_3$@IDOi@PEG@PEI (abbreviated as CaIP). Similarly, $CaCO_3$@PEG@PEI (abbreviated as CaP) nanoparticles were obtained by PEGylation and PEIylation of $CaCO_3$.

5 mg of the above CaIP was stirred with 3OD CpG ODNs in aqueous solution for 12 h to obtain the immuno-nanoagent $CaCO_3$@IDOi@PEG@PEI@CpG (abbreviated as CaIPC). As a control, the CaP particles were modified with CpG ODNs to obtain

$CaCO_3$@PEG@PEI@CpG (abbreviated as CaPC). The prepared nanoparticles were centrifuged at 8000 rpm for 10 min and redispersed in water for further use.

Preparation of liposome@IDOi@PEG@PEI@CpG (LIPC).

For comparison, liposomes loaded with IDOi were prepared by mixing a chloroform solution of DPPC, cholesterol, and DSPE-PEG at a 4:4:2 M ratio with IDOi under vigorous stirring overnight. Afterwards, the chloroform was evaporated, and the obtained liposome@IDOi@PEG nanoparticles were dispersed in aqueous solution for further use. The obtained nanoparticles were stirred with PEI for 24 h to prepare liposome@IDOi@PEG@PEI (abbreviated as LIP). Similarly, the liposome@IDOi@PEG@PEI nanoparticles were stirred with CpG ODNs in aqueous solution for 12 h to obtain liposome@IDOi@PEG@PEI@CpG (abbreviated as LIPC) as another control.

The release profiles of Ca^{2+}, IDOi and CpG ODNs.

The release profiles of Ca^{2+}, IDOi and CpG ODNs in pH 7.4 and pH 6.5 was studied by ICP-AES, UV/VIS spectrophotometer, and fluorescence spectrophotometer, respectively.

***In vivo* experiments.**

For the xenografts established from cultured cells, 4T1-Luc cells were suspended and harvested after trypsinization, and approximately 5×10^5 4T1-Luc cells in 150 μL of serum-free RPMI 1640 medium were subcutaneously injected into the right flank of the mice. The tumor volume (V) was determined by measuring the length (L) and width (W) and was calculated as $L \times W^2/2$.

To study the antitumor efficacy, mice with tumors were randomly divided into six groups (n = 8) and subjected to different treatments: I, normal saline (NS); II, $CaCO_3$@PEG@PEI (CaP); III, $CaCO_3$@IDOi@PEG@PEI (CaIP); IV, $CaCO_3$@PEG@PEI@CpG (CaPC); V, liposome@IDOi@PEG@PEI@CpG (LIPC); and VI, $CaCO_3$@IDOi@PEG@PEI@CpG (CaIPC). The intravenously injected dose was 50 mg/kg each time for 5 times in total. The tumor volumes and body weights of the mice were measured every other day for 14 days. Haematoxylin and eosin (H&E) staining of the five major organs (heart, liver, spleen, lung and kidney) was carried out at 12 h and 24 h

post-treatment to prove that the body's immune response resulted in minimal side effects to major organs.

To establish the long-term immune memory effect induced by CaIPC, the mice were subjected to two tumor inoculations. For the first tumor inoculation, 4T1-Luc cells (5×10^5) were subcutaneously injected into the left flank of the mice. After 5 days, the mice with tumors were randomly divided into six groups (n = 8) and subjected to different treatments: I, NS; II, CaP; III, CaIP; IV, CaPC; V, LIPC; and VI, CaIPC. The intravenously injected dose was also 50 mg/kg each time for five times total. Then, the tumors were removed by immunotherapy or surgery at day 20. For the second tumor inoculation, 4T1-Luc cells (5×10^5) were subcutaneously injected into the right flank of the mice at day 45, and the mice were injected with IDOi (1mg kg^{-1}) at day 47 and day 49. The tumor volumes and body weights of the mice were measured every other day for another 14 days.

To further establish the antitumor performance of CaIPC regarding recurrence, we next used the 4T1-Luc mouse breast cancer incomplete tumor resection model. Mice were randomly divided into six groups (n = 8) and subjected to different treatments after tumors were incompletely resected, leaving behind ~ 1% residual tissue: I, NS; II, CaP; III, CaIP; IV, CaPC; V, LIPC; and VI, CaIPC. Next, the mice in each group were intravenously injected with 50 mg/kg, 5 times in total. The tumor volumes of the mice were measured after 12 days. For comparison, another group of mice were peritumorally injected with 50 mg/kg in each group, 5 times in total.

***In vivo* bioluminescence and imaging.**

Bioluminescence images were collected with an *in vivo* imaging system (IVIS). The Living Image software (Xenogen) was used to acquire the data 10 min after the intraperitoneal injection of d-luciferin in DPBS (15 mg/mL) into the animals (10 µL/g of body weight).

ELISA analysis.

Serum cytokine levels were determined by enzyme-linked immunosorbent assays (ELISAs) using antibody pairs specific to these cytokines, following protocols

recommended by the manufacturer. Mice with tumors were divided into six groups (n = 8) and subjected to different treatments for five times: I, NS; II, CaP; III, CaIP; IV, CaPC; V, LIPC; and VI, CaIPC. After 12 h, the mice were sacrificed to harvest serum. Serum levels of IL-12 and IL-6 were determined with the Mouse IL-12 Mini ELISA Kit and Mouse IL-6 ELISA Kit, respectively.

Flow cytometry.

To analyse the different groups of immune cells, lymph nodes or tumors were harvested from mice in the different groups and stained with anti-CD3-APC, anti-CD8-PerCP, anti-CD44-FITC, anti-CD62L-PE, anti-CD4-FITC, and anti-Foxp3-PE antibodies according to the manufacturer's protocols. In brief, the lymph nodes or tumors were decomposed into smaller chunks and placed into a glass homogenizer containing PBS buffer (pH 7.4) with 2% heat-inactivated FBS. Next, a single cell suspension was obtained by gentle pressure with the homogenizer without adding digestive enzyme. Then, red blood cells (RBCs) in the single cell suspension were removed with RBC lysis buffer. Finally, the obtained single cells were stained with fluorescence-labelled antibodies. Central memory T cells (T_{CM}) and effector memory T cells (T_{EM}) were CD3＋CD8＋CD62L＋CD44＋ and CD3＋CD8＋CD62L－CD44＋, respectively. Tumor infiltration regulates T cells (Tregs) were CD3+CD4+Foxp3+, and CD8+ T cells were CD3+CD8+. All the antibodies in our experiments were used following protocols recommended by the manufacturer.

Mitochondrial oxygen consumption rate (OCR) of lymphocytes.

The OCR is an important indicator of cellular metabolism and function. In addition, a high OCR indicates a high metabolic rate and good cell viability. To analyse the impact of Ca^{2+} on lymphocytes, the OCRs of lymphocytes were studied. The lymphocytes were harvested from the lymph nodes of mice, divided randomly into three groups (n = 3) and subjected to different treatments: i, without treatment; ii, RPMI 1640 with 4 mM Ca^{2+}; and iii, RPMI 1640 with 50 μM BAPTA-AM (a chelator of intracellular Ca^{2+}). The mitochondrial OCR of the lymphocytes was measured 6 h later.

Statistical analysis.

All values in the present study are expressed as means ± SD. The significance between two groups was analysed by a two-tailed Student *t*-test. *P* value of less than 0.05 was considered significant (***P<0.001, **P<0.01, *P<0.05).

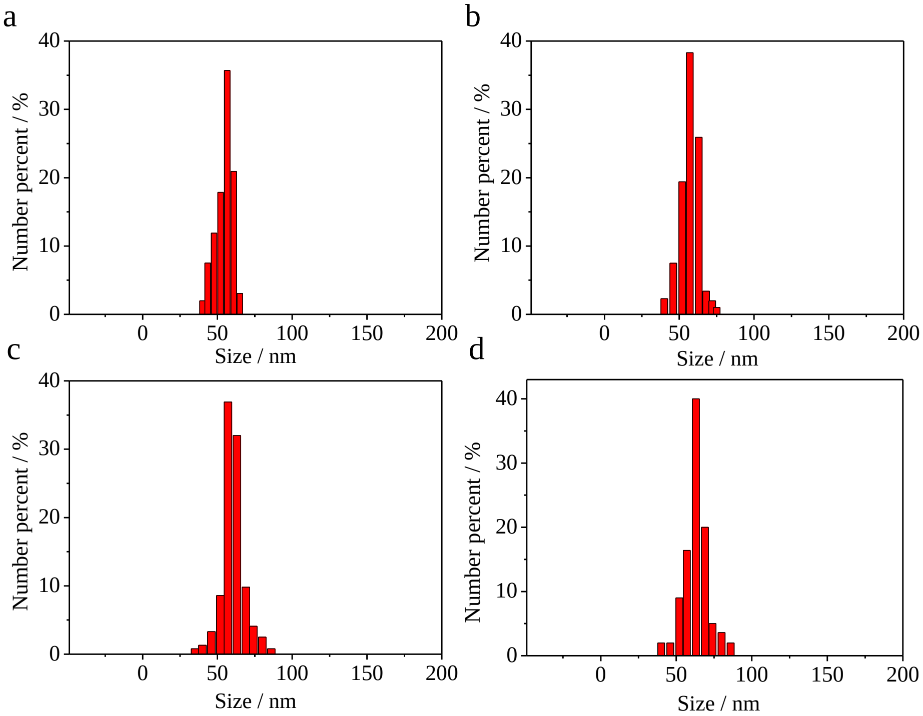

Figure S1 Size distribution of $CaCO_3$ (a), $CaCO_3$@IDOi@PEG (b), and $CaCO_3$@IDOi@PEG@PEI (c), $CaCO_3$@IDOi@PEG@PEI@CpG (CaIPC) (d).

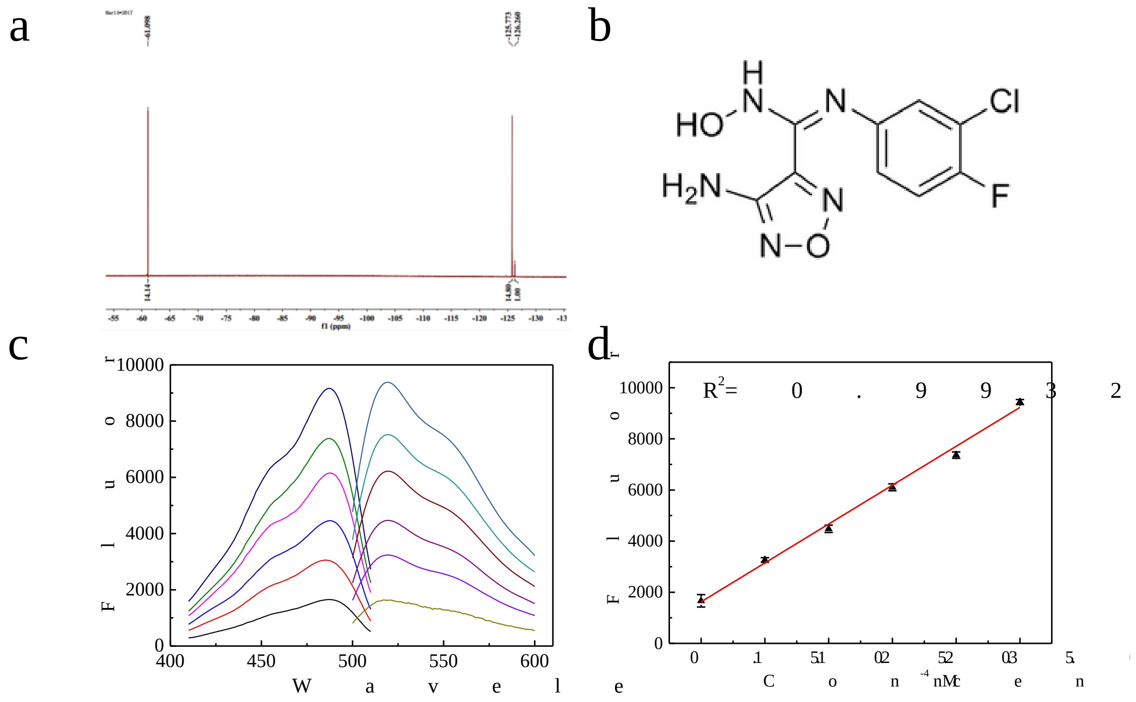

Figure S2 ^{19}F NMR spectrum of IDOi (a). Structural formula of IDOi (b). The excitation and emission spectra of CpG-FAM with different concentrations (c). Standard linear calibration curve of CpG-FAM (d).

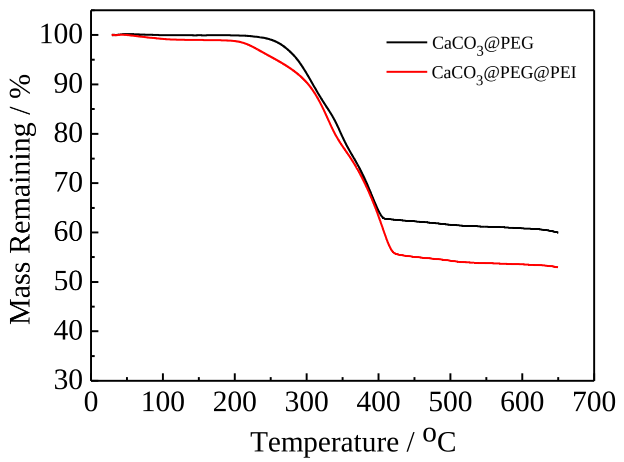

Figure S3 TGA of $CaCO_3$ with (red) or without PEI (black).

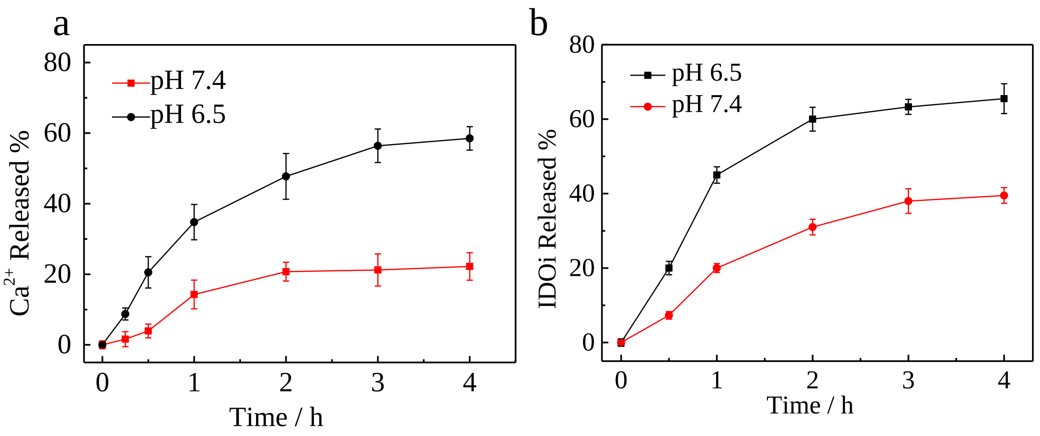

Figure S4 The release profiles of Ca^{2+} and IDOi (pH 6.5 and 7.4).

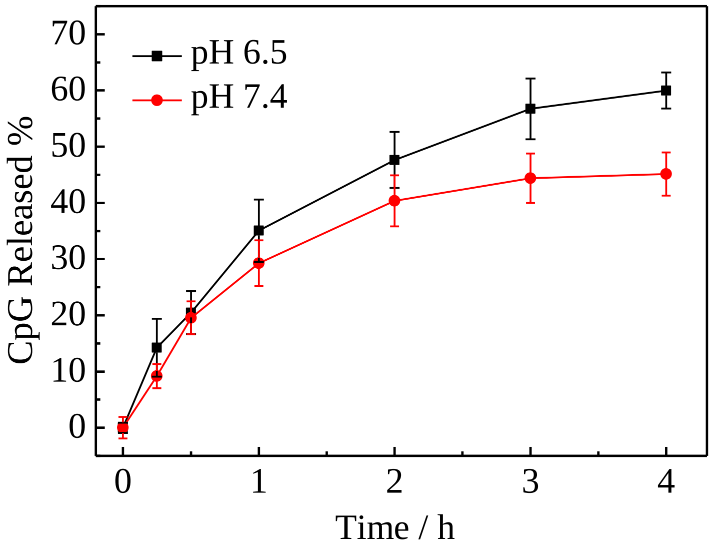

Figure S5 The release profiles of CpG (pH 6.5 and 7.4).

a b

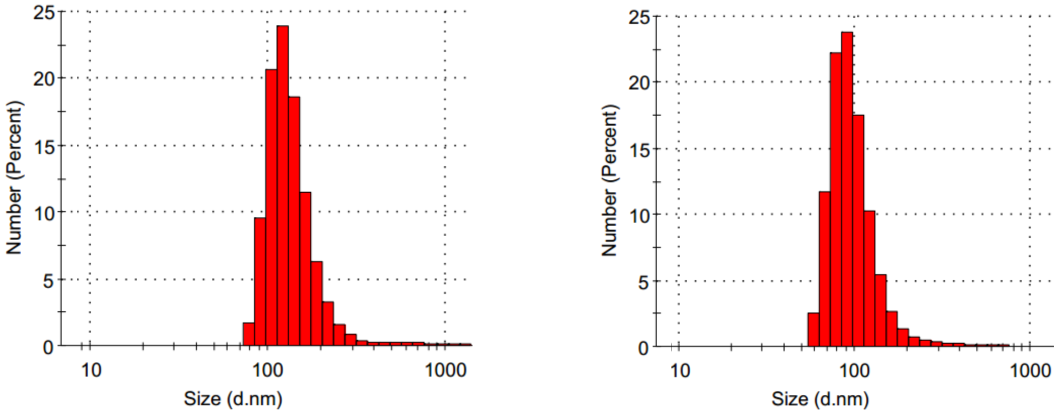

Figure S6 Particle size of CaIPC (a) and LIPC (b) as determined by dynamic light scattering (DLS).

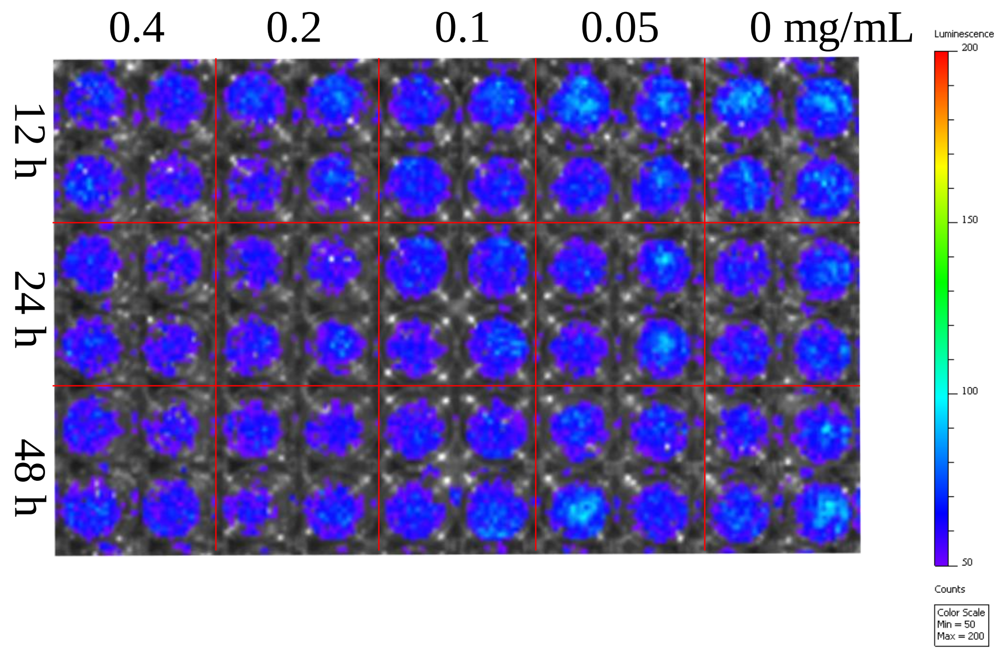

Figure S7 Bioluminescence images of 4T1-Luc cells incubated with 0, 0.05, 0.1, 0.2, or 0.4 mg/mL CaIPC for 12, 24 and 48 h.

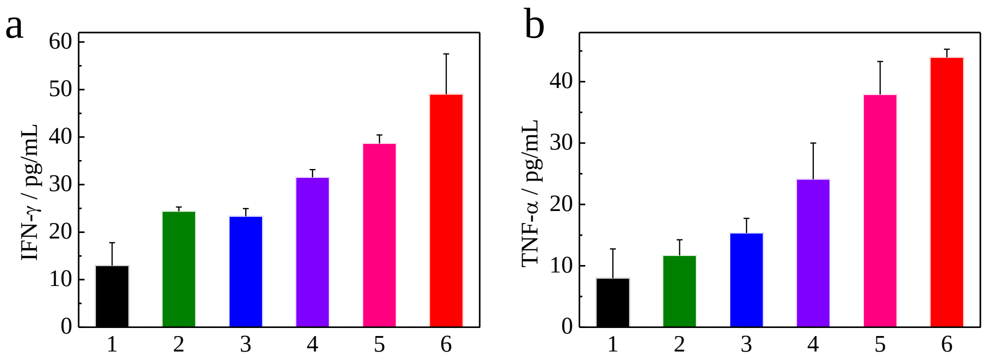

Figure S8 Serum IFN-γ (a) and TNF-α (b) levels of mice treated for 12 h with 1, NS; 2, CaP; 3, CaIP; 4, CaPC; 5, LIPC; and 6, CaIPC.

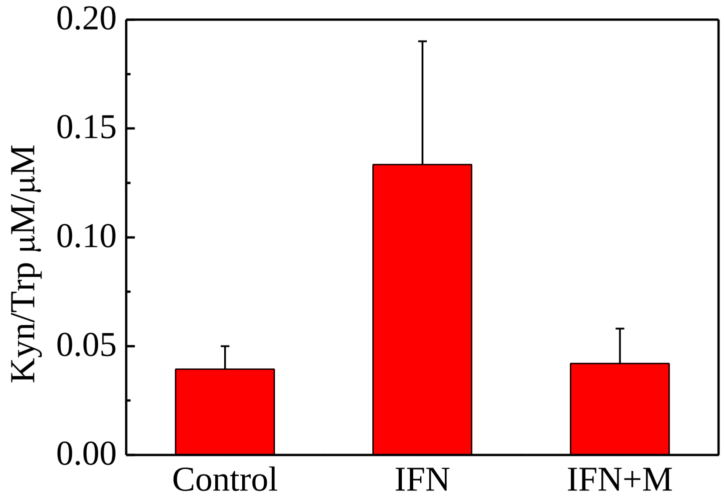

Figure S9 The ratio of kynurenine (Kyn) to tryptophan (Trp) in the cell culture medium.

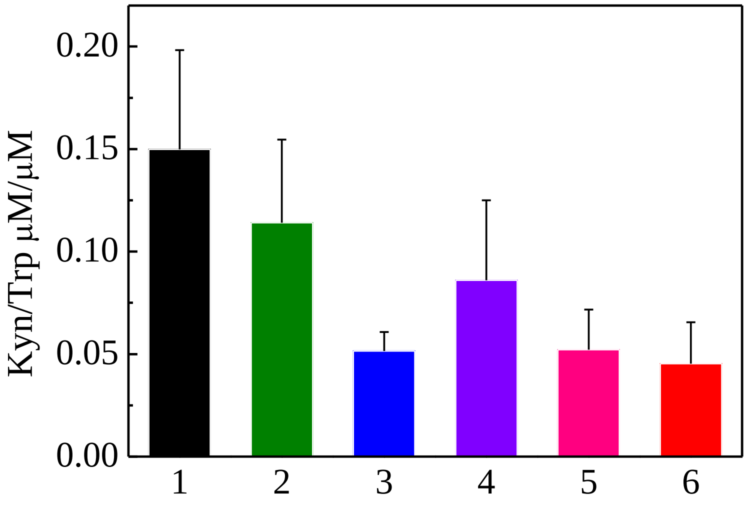

Figure S10 The ratio of kynurenine (Kyn) to tryptophan (Trp) in the tumors of mice. The mice were treated with 1, NS; 2, CaP; 3, CaIP; 4, CaPC; 5, LIPC; and 6, CaIPC.

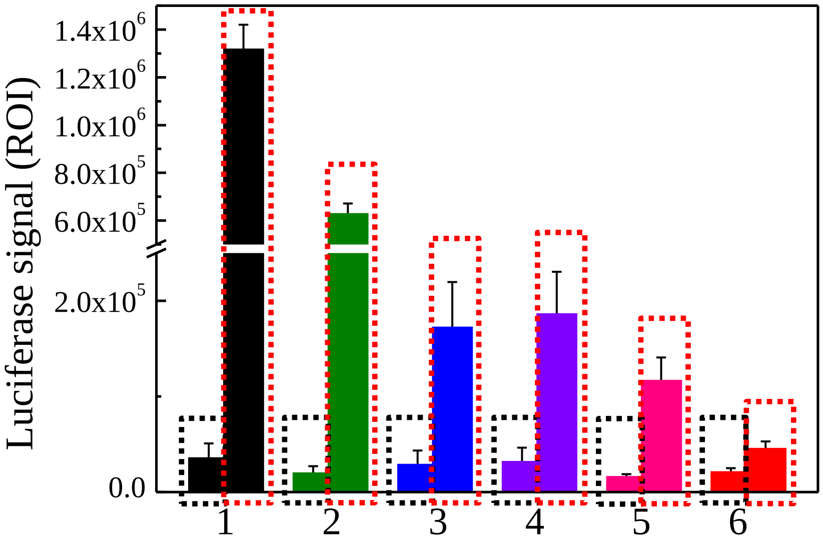

Figure S11 Corresponding bioluminescence quantification of tumors at day 0 (black dotted box) and day 14 (red dotted box).

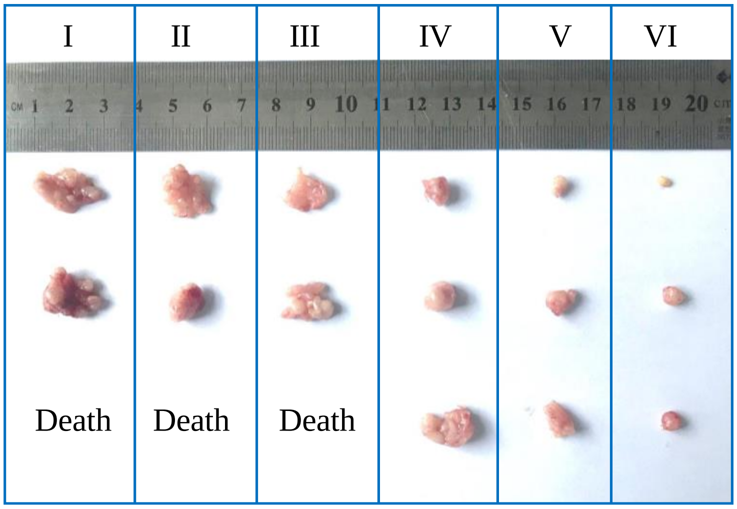

Figure S12 The therapeutic trials with our nanoagent in orthotopic mouse 4T1 tumor models. The mice were treated as follows: I, NS; II, CaP; III, CaPC; IV, CaIP; V, LIPC; and VI, CaIPC.

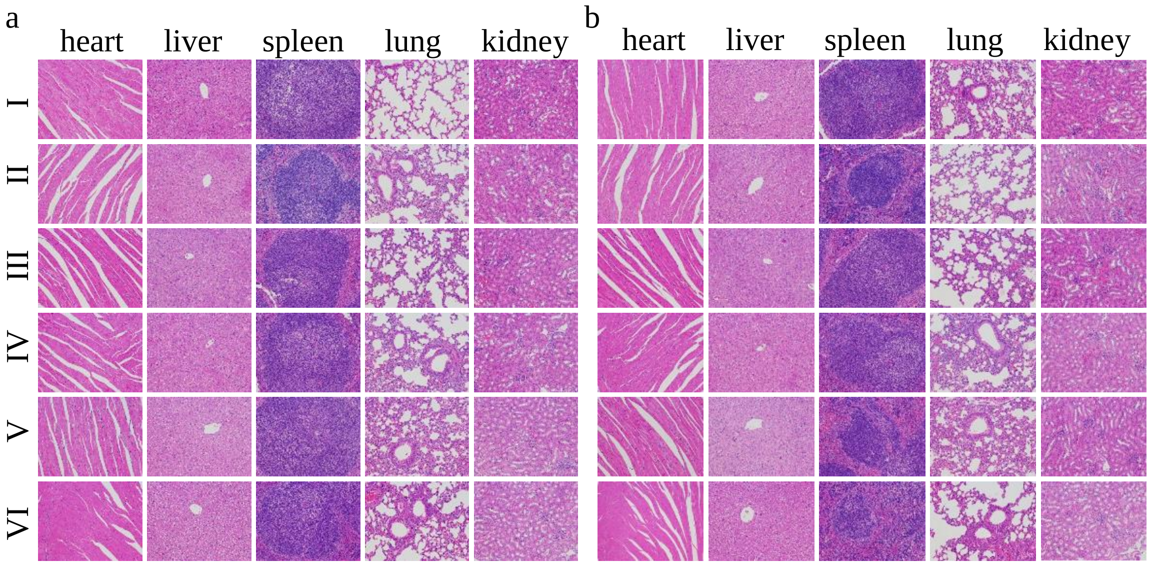

Figure S13 H&E staining of the five major organs (heart, liver, spleen, lung and kidney). The mice were treated as follows: I, NS; II, CaP; III, CaPC; IV, CaIP; V, LIPC; and VI, CaIPC, and H&E staining of tissue slides was carried out at 12 h (a) and 24 h (b) post-treatment.

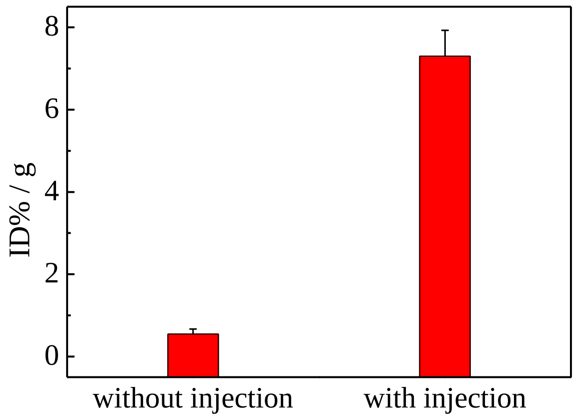

Figure S14 Tumor accumulation of nanoagent after injected for 24 h.

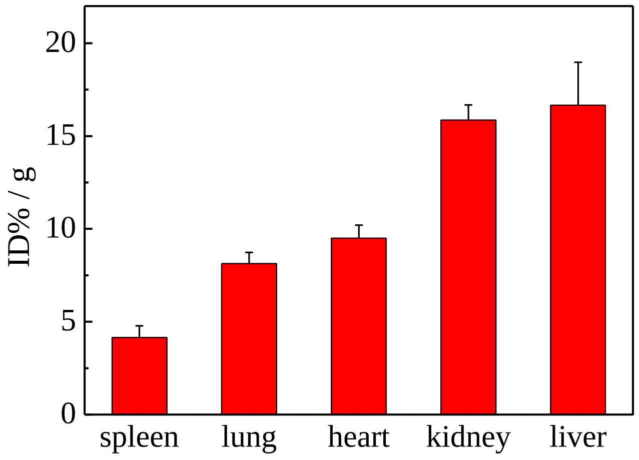

Figure S15 The distribution of nanoagent in liver, kidney, heart, lung, spleen by ICP-AES.

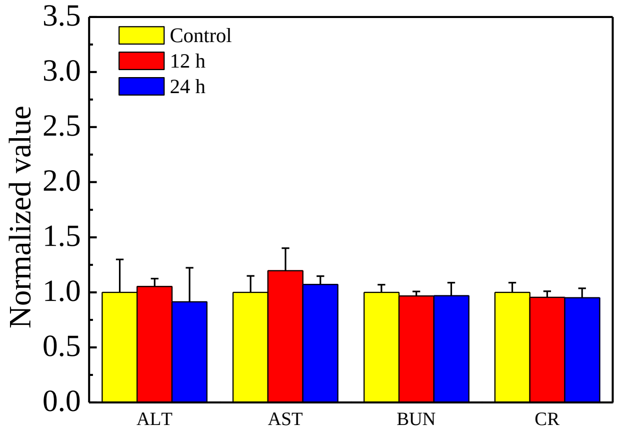

Figure S16 Blood biochemical tests of mice without treatment (Control) and with CaIPC for 12 h and 24 h.

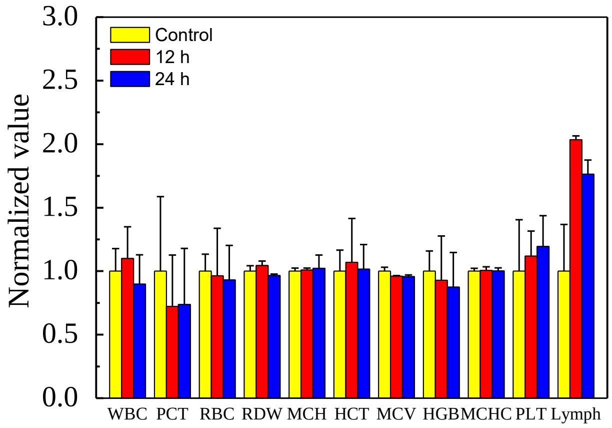

Figure S17 Blood routine tests of mice without treatment (Control) and with CaIPC for 12 h and 24 h.

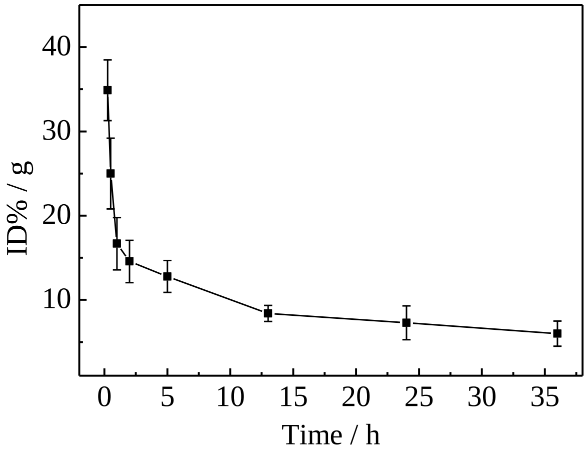

Figure S18 *In vivo* pharmacokinetic curve during 36 h after intravenously injected with nanoagent.

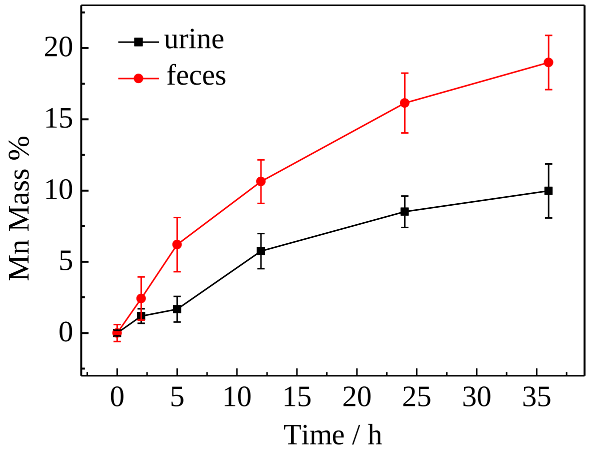

Figure S19 Mn amount in urine and feces of mice at various time points after injection.

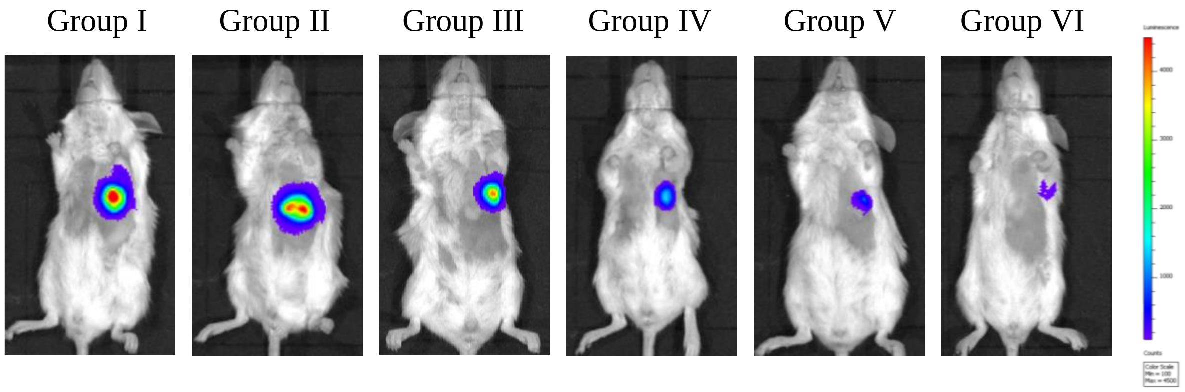

Figure S20 Bioluminescence images of mice with tumors from a rechallenge. Images of the mice were taken at day 61 with different treatments before elimination of the first tumors: I, NS; II, CaP; III, CaIP; IV, CaPC; V, LIPC; and VI, CaIPC. Images were acquired with the Living Image software (Xenogen) 10 min after intraperitoneal injection of d-luciferin (15 mg/mL) into the animals (10 μL/g of body weight).

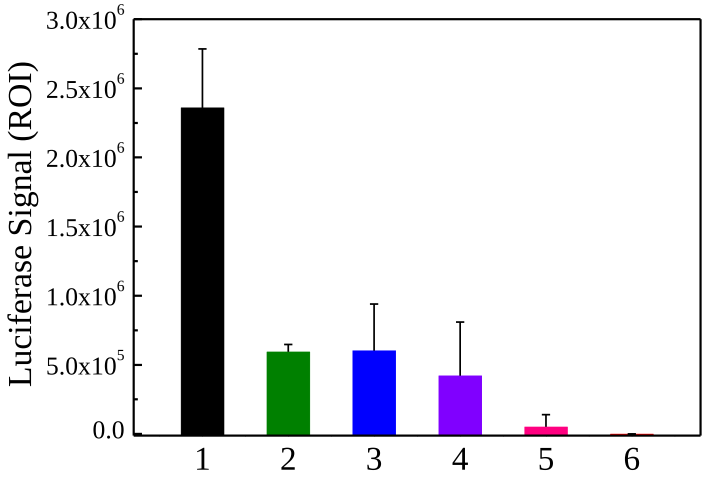

Figure S21 Corresponding bioluminescence quantification of tumors at day 12 for the different groups of mice after various treatments.

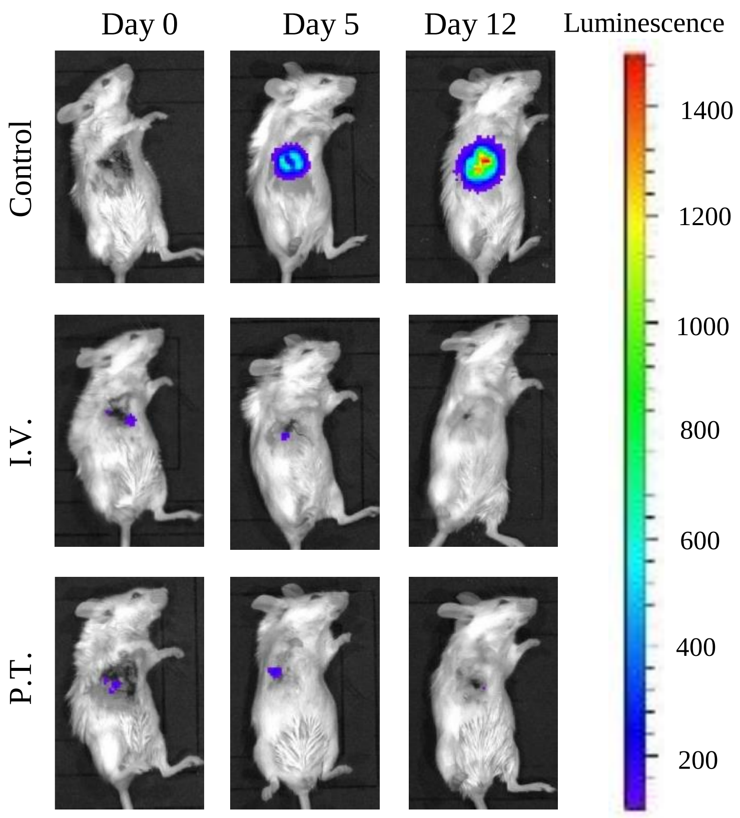

Figure S22 Tumor recurrence prevention by CaIPC via intravenous injection (I.V.) and peritumoral injection (P.T.).
